# Transactivation of Genes Encoding for Phase II Enzymes and Phase III Transporters by Phytochemical Antioxidants

**DOI:** 10.3390/molecules15096332

**Published:** 2010-09-07

**Authors:** Yoon Mee Yang, Kyoung Noh, Chang Yeob Han, Sang Geon Kim

**Affiliations:** Innovative Drug Research Center for Metabolic and Inflammatory Disease, College of Pharmacy, Seoul National University, Gwanak-ro 599, Seoul 151-742, Korea

**Keywords:** phase 2 enzyme, phase 3 transporter, NF-E2-related factor 2, CCAAT-enhancer binding protein-β, hepatic nuclear factor, farnesoid X receptor

## Abstract

The induction of phase II enzymes and phase III transporters contributes to the metabolism, detoxification of xenobiotics, antioxidant capacity, redox homeostasis and cell viability. Transactivation of the genes that encode for phase II enzymes and phase III transporters is coordinatively regulated by activating transcription factors in response to external stimuli. Comprehensive studies indicate that antioxidant phytochemicals promote the induction of phase II enzymes and/or phase III transporters through various signaling pathways, including phosphoinositide 3-kinase, protein kinase C, and mitogen-activated protein kinases. This paper focuses on the molecular mechanisms and signaling pathways responsible for the transactivation of genes encoding for these proteins, as orchestrated by a series of transcription factors and related signaling components.

## 1. Introduction

Biotransformation of xenobiotics including drugs is catalyzed by enzymes which are commonly referred to as drug-metabolizing enzymes. Most tissues and organs have detoxifying systems responsible for the transformation and removal of chemicals. Proteins, which include phase I, phase II enzymes and phase III transporters, play key roles in the metabolism, detoxification, and/or elimination of exogenous chemicals introduced into the body as well as endogenous ones [1,2]. The metabolizing enzymes are basally expressed and/or induced by external stimuli. In addition, diverse phytochemicals have beneficial actions by upregulating them.

### 1.1. Phase II enzyme induction

Phase II enzymes such as UDP-glucuronosyl transferases, glutathione *S*-transferases (GSTs), NAD(P)H:quinone oxidoreductases (NQOs), and *N*-acetyltransferases catalyze conjugation reactions of exogenous and endogenous chemicals, usually after phase I reactions (*i.e.*, oxidation, reduction and hydrolysis) [1]. In general, they mediate detoxification and elimination of toxicants through diverse reactions (e.g., glucuronidation, sulfation, acetylation and methylation), whereas phase I oxidation reactions may often produce reactive metabolites. If the conjugation reactions are inadequate, active metabolites may cause damage and injury to cells and tissues, which is frequently accompanied by inflammatory responses [3]. Thus, the inducers of phase II enzymes have cytoprotective effects.

### 1.2. Phase III transporter induction

Phase III transporters are expressed in many tissues, including the liver, intestine, kidney and brain, where they provide a barrier against drug penetration, acting as the major determinants of the systemic bioavailability of many drugs (e.g., absorption, distribution and excretion) [4,5]. *P*-glycoprotein (P-gp) and multidrug resistant-associated protein (MRP) transport a broad range of substrates across the cell membrane by utilizing the energy released from ATP hydrolysis [4]. The ATP-binding cassette (ABC) transporters either import or export various substrates such as sugars, amino acids, lipids, ions, xenobiotics and many therapeutic agents [6,7]; they have two nucleotide binding domains and two transmembrane domains. The nucleotide binding domain, also called as an ABC, is the main characteristic of transporter family, and the transmembrane domain facilitates the movement of substrates across the cell membranes [6,7].

In humans, 46 ABC transporters have been identified [4,6]. Along with P-gp (or MDR1; ABCB1), the MDR subfamily includes MDR3 (ABCB4), and bile salt export protein (Bsep or SP-gp; ABCB11) [8]. The MRP subfamily consists of nine subfamilies (MRP1-9) [6]. MRP1 (ABCC1) and MRP3 (ABCC3) are typically located on the basolateral membrane of polarized cells, whereas MRP2 (ABCC2) mostly exists in the apical canalicular membrane, implying that MRP2-mediated transport increases excretion into bile, whereas MRP1/3-mediated transport does it into the urine. Organic anion transporting polypeptide 2 (OATP2), localized in the hepatic sinusoidal membrane, mediates ATP- and sodium-independent transport of compounds, including bilirubin, steroids, type II organic cations, thyroid hormones and bile salts [9,10]. P-gp, MRP and OATP2 that are expressed on the membrane of the intestinal enterocytes excrete xenobiotics into the lumen [7,11].

In response to various extracellular stimuli, the transactivation of phase II enzyme and phase III transporter genes is coordinately regulated by activating transcription factors. This paper focuses on the molecular mechanisms of transcriptional induction of the genes orchestrated by a series of transcriptional factors.

## 2. Transcription Factors that Promote Phase II and Phase III Gene Induction

### 2.1. NF-E2-related factor 2 (Nrf2)

Nrf2 is a Cap‘n’Collar/basic leucine zipper transcription factor. In the resting state, Keap1 interacts with Nrf2 for its degradation by Cullin 3-mediated ubiquitination in the cytoplasm [12]. When Keap1 dissociates from Nrf2 under oxidative and xenobiotic stress, Nrf2 is phosphorylated and translocated into the nucleus [13]. Unlike canonical bZIP proteins, Nrf2 can not form homodimer [14]. It forms a heterodimer with small Maf proteins (e.g., MafF/G/K) that lack a canonical transactivation domain.

The induction of phase II enzymes and phase III transporters depends on the activity of Nrf2. Major anti-oxidant enzymes contain one or more functional antioxidant response elements (AREs) in their promoter regions. Once Nrf2 dissociates from its Keap1 binding in response to oxidative stress, it translocates into the nucleus and binds ARE in the target genes. Thus, Nrf2 activation transactivates the genes containing ARE(s) such as GST, heme oxygenases-1 (HO-1), UDP-glucuronosyl transferase, NQO-1, γ-glutamylcysteine synthetase and organic anion transporters [12]. Nrf2 activation and gene induction contribute to the detoxification and excretion of xenobiotics. Plant or synthetic chemicals may have cytoprotective and chemopreventive effects by activating Nrf2 [15]. Consistently, a deficiency in Nrf2 abrogates the abilities of these agents to protect cells from toxicant or other stresses.

### 2.2. CCAAT-enhancer binding protein-β (C/EBPβ)

Among the members of C/EBP family, C/EBPβ is a transcription factor responsible for the expression of genes encoding for antioxidant and/or conjugating enzymes [16]. It binds to a C/EBP-binding site as homo- or heterodimers. The localization and/or activity of C/EBPβ can be regulated by phosphorylation. C/EBPβ is phosphorylated by p90 ribosomal S6 kinase-1 (RSK1) and translocated from the cytoplasm into the nucleus [17]. Once in the nucleus, a phosphorylated form binds the C/EBP-response element [18]. Ceramide, a toxic lipid, decreased the transcriptional activity of C/EBPβ by reducing its phosphorylation [19]. In contrast, treatment of hepatocytes with oltipraz, a cancer chemopreventive agent, activated and induced C/EBPβ. The activation of C/EBPβ led to phase II enzyme induction, contributing to its antioxidant effect [20]. Prostaglandin J_2_ treatment also induces GSTA2 by activating C/EBPβ as well as Nrf2 [21]. In most cases, the expression of phase II genes may be coordinately regulated by C/EBPβ and Nrf2 that make a large enhanceosome complex.

### 2.3. Hepatic nuclear factor 1 (HNF1)

HNF1, a liver-specific gene transactivator, is a dimeric transcription regulator. HNF1α, but not HNF1β, exists in hepatocytes [22] and is also expressed in other tissues including kidney, intestine and pancreatic islets [23]. The binding of HNF1α to the *cis*-acting HNF1-binding element in the target promoters regulates the expression of genes including glucose-6-phosphatase, albumin, α-lipoproteinAII and B, and CYP2E1 [22,24]. The transcriptional activity of HNFs is regulated by coactivators such as CBP, p300 and p300/CBP-associated factor [22]. Several reports have suggested the important role of HNF1 in cell survival. So, HNF1α deficiency causes hepatic dysfunction [25]; inhibition of HNF1α triggers mitochondrial hyperpolarization and apoptotic cell death in response to toxic stimuli (e.g., ceramide) [26]. For example, ceramide enhances the degradation of HNF1 [27], which might cause apoptosis. Furthermore, HNF recognition element has been identified in the promoter region of the *GSTA2* gene [28]. Oltipraz treatment increased the nuclear accumulation and DNA binding of HNF1 [27], indicating that the activation of HNF1 might contribute to its cytoprotective effect. HNF1α is a master regulator of several transporter families. HNF1α disruption results in significant downregulation of several organic anion transporters (Oat) and Oatp uptake transporters in liver and kidney, but increases the expression of efflux transporters (e.g., MDR and MRP) [29].

Liver-enriched HNF4α promotes the expression of genes involved in hepatic lipid homeostasis and hepatocyte differentiation [30,31]. It also regulates the expression of phase II enzymes and phase III transporters (e.g., UGT1A9) [32]. In addition, HNF4α, not HNF1α, binds to the Ntcp promoter. The HNF4α binding site located in the human steroid- and bile acid-sulfotransferase gene enhances basal promoter activity [33]. In fasted rats, HNF4α upregulates the basolateral bile acid transporters (e.g., Ntcp, Oatp1, and Oatp2) [34].

### 2.4. Peroxisome proliferator-activated receptors (PPARs)

Currently, three members of this nuclear receptor family have been identified, namely PPARα, PPARβ and PPARγ [35,36]. PPARα is expressed in the liver, heart, kidney, intestine and brown adipose tissue. PPARβ is expressed in most adult tissues; brain, kidney and intestine are the highest expressed tissues. PPARγ, mainly expressed in the spleen, intestine and fat cells, is composed of two submembers, PPARγ1 and PPARγ2. PPARs regulate physiological functions such as lipoprotein and fatty acid metabolism [1,2,36,37,38]. In the *GSTA2* gene, a PPAR-binding site cluster was identified.In particular, specific mutations in the peroxisome proliferator response element (PPRE) sites caused defect in the responsiveness [21]. PPARγ and retinoid X receptor (RXR) activate the *GSTA2* gene [21]. In addition, the PPARγ agonist and 9-*cis* retinoic acid synergistically enhanced the activities of Nrf2 and C/EBPβ [21].

### 2.5. Nuclear receptors [pregnane X receptor (PXR), farnesoid X receptor (FXR)]

The expression of phase III transporters such as P-gp, depends on PXR [39,40]. PXR ligands including rifampicin, clotrimazole, mifepristone and nifedipine induced *MDR1* gene in hepatocytes and cancer cells [2,41,42]. Constitutively activated hPXR also induces P-gp without specific ligand binding. A direct repeat 4 nuclear receptor response element was identified as a distinct PXR binding site essential for *MDR1* induction by rifampin [43]. In addition, PXR activation causes the induction of other transporters including OATP2 [44,45], MRP2 [46] and MRP3 [47,48].

FXR (NR1H4) is expressed in liver, intestine, kidney and adrenal glands [49,50,51]. Bile acids including chenodeoxycholic acid are endogenous ligands of the receptor [52]. FXR has diverse physiological roles in the regulation of bile acid, lipid and glucose metabolism. As a transcription factor, it regulates the expression of genes including hepatic transporters; Bsep, MRP2 (ABCC2) and MDR3 (ABCB4) are present in the bile canalicular membrane and thereby help secrete bile acids (and other compounds) [53,54]. FXR also controls the process of bile acid absorption via apical sodium-dependent bile acid transporter, heterodimeric organic solute transporter-α and -β. Thus, FXR is a key sensor for bile acids and plays a role in maintaining bile acid homeostasis such as bile acid synthesis, conjugation, secretion and absorption.

### 2.6. Cooperative interactions of activating transcription factors

Diverse transcription factors cooperatively regulate the expression of phase II and/or phase III enzymes. The *GSTA2* gene transactivation is controlled by both the ARE and C/EBP-binding sites [21]. A deletion of either ARE or C/EBP-binding sites prevented the PPARγ and RXRα-mediated *GSTA2* gene induction, indicating that Nrf2 and C/EBPβ binding to their responsive DNA elements are essential for full transactivation of the gene. In addition, the ligand-dependent transcriptional activity was inhibited by a mutation of the respective PPRE binding site [21], suggesting that the PPREs are important for the full ligand responsiveness. Thus, protein complex formation on target DNA binding site seems to be an important step for transcriptional activation by inducers.

## 3. The Signaling Pathways for Transcription Factor Activation

### 3.1. Phosphatidylinositol 3-kinase (PI3K)

**Figure 1:**
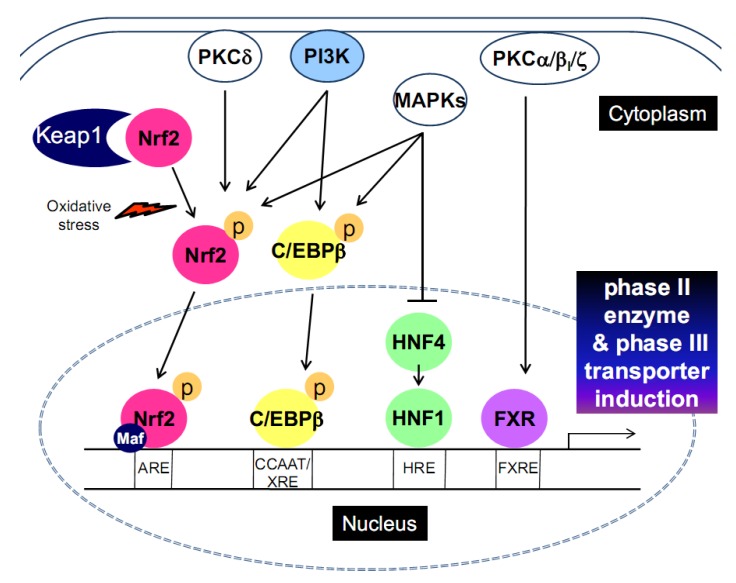
The signaling pathways for transcription factor activation that leads to phase II enzyme and phase III transporter induction.

PI3K phosphorylates phosphatidylinositols at the 3 position of the inositol ring, and the downstream Akt-p70S6 kinase pathway regulates a variety of biological responses including cell proliferation, survival, mitogenesis and cell transformation [55]. PI3K has been reported to act as a positive regulator of Nrf2 binding with ARE [56] (Figure 1). Kang *et al.* showed that PI3K is involved in nuclear localization of Nrf2 by *tert*-butylhydroquinone-induced oxidative stress, and is associated with cytoplasmic actin rearrangement [57].

Insulin stimulates Nrf2 activity and induces GSTA2 [57]. Since Akt and RSK1, the downstream molecules of PI3K, are activated by insulin, the induction of GSTA2 may depend on the activation of mTOR complex. The finding that ceramide decreased S6K1 activity and protein synthesis [58] indicates that ceramide inhibits GSTA2 expression [59] at least in part through the repression of the mTOR pathway. Thus, mTOR signaling may be involved in the regulation of GST expression. Insulin also activates C/EBPs via PI3K [60]. Likewise, α-lipoic acid treatment induced phase II enzymes through PI3K-dependent activation of C/EBPα and C/ΕΒPβ, enhancing the ability of insulin to induce target genes [20] (Figure 1). In addition, the activation of C/EBPβ by oltipraz and its metabolites contributes to the induction of phase II genes in a PI3K-dependent manner [18,61].

### 3.2. Protein kinase C (PKC)

PKCs consist of 12 isoforms of the PKC family, which are grouped into 3 subfamilies based on their second messenger requirements: 1) conventional (PKCα, PKCβ_I_, PKCβ_II_ and PKCγ), 2) novel (PKCδ, PKCε, PKCη and PKCθ) and 3) atypical (PKCι, PKCζ, PK-N1 and PK-N2). Conventional PKCs require diacylglycerol phospholipase C, Ca^2+^ and phospholipid for activation. Novel PKCs require diacylglycerol phospholipase, but not Ca^2+^. Atypical PKCs require neither diacylglycerol phospholipase nor Ca^2+^. In some cells, PKCζ may be at downstream of PI3K, whose activation depends on PI3K products [62].

Nrf2 activation requires phosphorylation at serine-40 by PKCδ [63,64] (Figure 1): a mutant form of Nrf2 (S40A) could not be phosphorylated by PKC. This mutation affects the association of Nrf2 with Keap1, but not the *in vitro* binding of Nrf2/MafK to the ARE [63,64]. The phosphorylation of wild-type Nrf2 by PKCδ promotes its dissociation from Keap1, contributing to its stabilization. This finding indicates that PKCδ-induced Nrf2 phosphorylation is crucial for ARE-mediated antioxidant response. Treatment with PKC activator, phorbol 12-myristate 13-acetate, increased the phosphorylation of FXR. A study showed that the DNA binding domain of FXR was *in vitro* phosphorylated by PKCα and PKCβ_I_ [65] (Figure 1). The phosphorylation of FXR induced by PKCα directly modulates ligand-mediated regulation of FXR target genes. Consistently, the induction of FXR target genes by chenodeoxycholic acid was repressed by PKC inhibition, but not by PKA or PI3K inhibition. In addition, PKCζ directly phosphorylates FXR at threonine 442 site. So, PKCζ knockdown decreased its nuclear localization [66].

### 3.3. Mitogen-activated protein kinases (MAPKs)

Three major MAPK pathways [*i.e.*, extracellular signal-regulated kinase (ERK), *c*-Jun N-terminal kinase (JNK) and p38 kinase] are involved in the regulation of many transcription factors, which affects the phase II enzyme and phase III transporter expression (Figure 1). Nrf2 activity may be modulated by MAPKs [67]; ERK2, ERK5 and JNK1 increase ARE activation [68,69,70], whereas p38 kinase suppresses it [71].

C/EBPβ is regulated by the MAPK pathways. Interferon γ-stimulated pathway stimulates C/EBPβ-dependent gene expression via MEKK-MEK1-ERK1/2 and p38 kinase [72,73]. In addition, JNK inhibition reduced C/EBPβ expression, indicating that phosphorylation induced by JNK participates in C/EBPβ expression [74].

The transcriptional activity of HNF4α may be regulated by post-translational modifications. Thirteen potential serine/threonine phosphorylation sites exist in HNF4α. It is phosphorylated by kinases including p38 kinase, ERK1/2, PKA, PKB, PKC and AMPK, and the phosphorylated forms have lower DNA binding, dimerization or transactivation activities [75,76]. JNK1 phosphorylates HNF4α, and reduces its interaction with DNA. Of interest, HNF1α negatively regulates its own and HNF4 expressions by a negative feedback loop [77]. HNF1α expression in turn depends on HNF4α expression, and is reduced under the condition of reduced HNF4α activity [78,79,80].

The members of the RSK family play a role in mitogen-activated cell growth, differentiation, or cell survival. RSK1 is a major form expressed in the liver, muscle and fat [81]. RSK, a serine/threonine protein kinase, is activated by ERK [82]. It contains two distinct active kinase domains. Activated RSK1 phosphorylates C/EBPβ and CREB [83].

## 4. The Induction of Phase II Enzymes and/or Phase III Transporters by Antioxidant Phytochemicals

### 4.1. Genistein

Genistein, a biologically active isoflavone found in soy, has a chemopreventive effect. Genistein modulates the expression of genes encoding for phase II and antioxidant enzymes. Feeding rats with diets containing genistein stimulated hepatic NQO-1 activity. It increased hepatic GSTA2 mRNA level, but decreased those of GSTM2 and GSTP1 [84]. However, GST activity was decreased in the liver of mice fed 1,500 mg/kg of genistein [85]. Genistein treatment repressed sulfotransferase 1E1, UGT1A1, UGT2B7, UGT2B15, MRP2 and MRP4 mRNA levels [86].

### 4.2. Resveratrol

Resveratrol (3,5,4'-trihydroxy-*trans*-stilbene) is a natural polyphenol compound present in grapes and peanuts. This agent has a variety of potential therapeutic effects. Many of the beneficial effects of resveratrol are a result of its antioxidant action. Resveratrol scavenges not only lipid hydroperoxyl free radicals, but hydroxyl and superoxide anion radicals and thus, resveratrol treatment protects cells from oxidative stress by increasing Nrf2 activity through Akt/protein kinase B and ERK1/2 pathways [87].

Resveratrol alters the profile of xenobiotic-metabolizing enzyme activity; GST was significantly inhibited, particularly in the lung (~76% loss of activity) after single administration of 25 mg resveratrol/kg b.w. A different response for UDP-glucuronosyl transferase was observed; a significant induction was seen (83%) in the liver, whereas a significant reduction was observed in the lung (up to ~83% loss) after treatment with 25 mg resveratrol/kg b.w. for 7 days [88]. Resveratrol also regulates the expression of phase III transporters; it down-regulates MRP1 expression and thereby reverses doxorubicin resistance in acute myeloid leukemia cells [89].

### 4.3. Liquiritigenin

**Figure 2:**
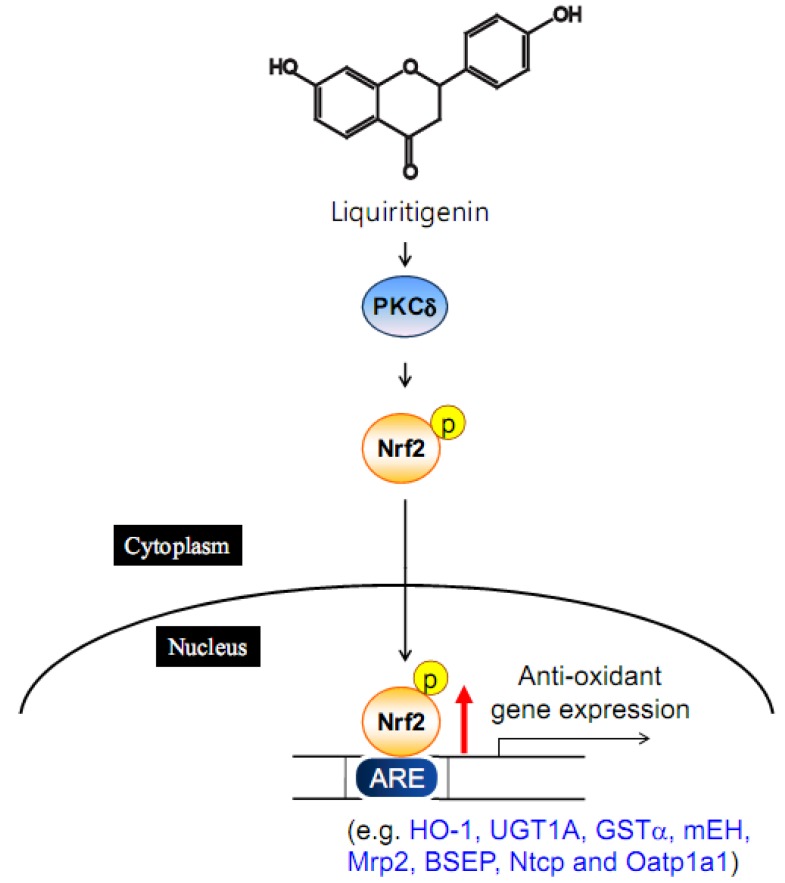
The induction of phase II enzyme and phase III transporters by liquiritigenin.

Liquiritigenin, a biologically active licorice component, inhibited LPS-induced NO synthase induction [90]. After intravenous administration of liquiritigenin, bile flow rate and biliary excretion of bile acid, glutathione and bilirubin contents were elevated [91]. Liquiritigenin treatment markedly stimulated Nrf2 translocation into the nucleus via PKCδ activation [92]. The natural compound enhances not only the expression of hepatic phase II enzymes but that of canalicular efflux transporters and basolateral uptake transporters [91] (Figure 2). Consistently, liquiritigenin treatment attenuated galactosamine/LPS-induced hepatitis in rats [91]. Overall, liquiritigenin has a hepatoprotective effect by inducing phase II enzymes and phase III transporters.

### 4.4. Sauchinone

Sauchinone, a lignan from the roots of *Saururus chinensis* (Lour.) Baill, Saururaceae, has potent hepatoprotective and anti-inflammatory actions [93,94]. It also inhibits bone resorption [95]. In a cell culture model, sauchinone treatment induced HO-1 expression and activity, which in part accounts for its cytoprotective efficacy against oxidative injury [96]. Moreover, sauchinone enhanced nuclear accumulation of Nrf2, and increased ARE activity. Sauchinone protects cells from *t*-butyl-hydroperoxide-induced oxidative injury, possibly through p38 kinase-mediated Nrf2/ARE-dependent HO-1 induction [96].

### 4.5. Dithiolethiones

Oltipraz [4-methyl-5-(2-pyrazinyl)-1,2-dithiole-3-thione] is a synthetic compound that has the dithiolethione moiety found in the *Crucifera.* [97]. Dithiolethiones and some of their metabolites are inducers of genes encoding for phase II enzymes [18,61,98,99]. Oltipraz exerts its cancer chemopreventive effect by inducing these enzymes [100,101]. Nrf2 is critical for the enzyme induction by oltipraz [98,102,103]. Also, oltipraz induced C/EBPβ [18,83], and antagonized the effect of hepatitis B virus X that represses C/EBPβ-mediated GST induction [104]. Phase III transporters are also induced by oltipraz treatment [105,106]. The effect of oltipraz on MRP expression is mediated with Nrf2 [105,106]. Thus, oltipraz’s actions seem to be cooperatively regulated by both Nrf2 and C/EBPβ.

## 5. Conclusions

**Figure 3:**
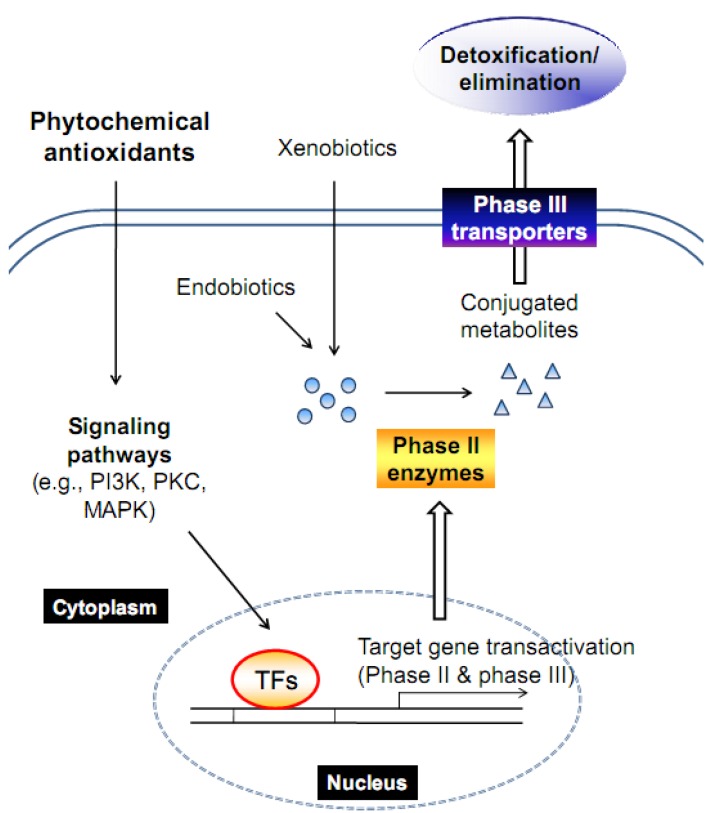
A schematic representation of the mechanism by which phytochemical induces target genes.

Living organisms have their own defense mechanisms to protect themselves from cellular damage caused by oxidative stress. The ability of cells to maintain homeostasis during stress can be achieved by inducing detoxifying enzymes and transporters and consequently removing harmful substances. A battery of genes encoding for these proteins shares common transcriptional regulatory mechanism (Figure 3). Antioxidant phytochemicals activate signaling pathways responsible for the regulation of key transcription factors, thereby inducing phase II and phase III proteins for the improved metabolism and excretion of xenobiotics.
